# Stress Relaxation and Creep of a Polymer-Aluminum Composite Produced through Selective Laser Sintering

**DOI:** 10.3390/polym12040830

**Published:** 2020-04-05

**Authors:** Jerzy Bochnia, Slawomir Blasiak

**Affiliations:** Department of Manufacturing Engineering and Metrology, Faculty of Mechatronics and Mechanical Engineering, Kielce University of Technology, 25-314 Kielce, Poland; j.bochnia@tu.kielce.pl

**Keywords:** additive technology, polymer materials, stress relaxation, creep

## Abstract

This article discusses the rheological properties (stress relaxation and creep) of polymer-aluminum composite specimens fabricated through the selective laser sintering (SLS) from a commercially available powder called Alumide. The rheological data predicted using the Maxwell–Wiechert and the Kelvin–Voigt models for stress relaxation and creep, respectively, were in agreement with the experimental results. The elastic moduli and dynamic viscosities were determined with high accuracy for both models. The findings of this study can be useful to designers and users of SLS prints made from the material tested.

## 1. Introduction

Three-dimensional printing or additive manufacturing, originally known as Rapid Prototyping, is being increasingly used in various applications, including manufacturing, design, architecture and medicine [1,2,3]. 3D Printing has become a common process to fabricate not only models, patterns or prototypes, but also finished and semi-finished products. The latest developments in additive manufacturing, especially its application to medical devices and pharmaceuticals, have been discussed, for instance, in [4,5]. The articles analyze the current and future areas of use of 3D printing in manufacturing as well as recent advances in materials and designs.

The most important factors affecting the development of additive manufacturing are:

− The ability to customize products according to individual needs and requirements.
− The ability to design and fabricate elements complex in shape, which are impossible or very difficult to produce using conventional processes.
− The ability to combine many components into one single product to save assembly costs.

Additive manufacturing technologies are suitable to print geometrically intricate internal structures, e.g., objects within objects, thin-walled products, or objects with a sponge, cellular or honeycomb structure retaining sufficient strength. Fused deposition modelling (FDM) is the most popular 3D printing process for quick and easy fabrication [6,7]. In medicine, for example, FDM technology has been used for hip joint reconstruction. Pre-surgical modelling involves simulating loads and calculating unit stresses, and developing a custom implant design [8].

In additive manufacturing, the layer-by-layer deposition considerably affects the material mechanical properties [9,10,11,12]. This is crucial in the manufacture of consumer products, particularly those subjected to external loads, where high material strength is required.

The rheological properties of additively manufactured materials vary, depending on the build direction. The problem has been investigated extensively by many researchers, e.g., [13]. The rheological properties of materials are usually determined by comparing them to ideal solids. The stress relaxation behavior can be predicted by means of the Maxwell model [14,15,16,17,18]. There are various forms of the Maxwell model: simple and complex, for example, models based on Prony series or ones described by fractional calculus [19]. In the case of creep, curves can be described using different forms of the Kelvin and Kelvin–Voight models (models with two, three or more parameters) [20,21,22].

The application of plastics in additive manufacturing, especially Selective Laser Sintering (SLS), goes back to the early 1990s. Since then, this technology has developed and a wide range of materials differing in chemical composition and physical properties has been introduced. One of the strengths of SLS is that elements are almost immediately cooled to ambient temperature and ready to use. Before the method is applied on an industrial scale, extensive research into the physical properties of materials is required.

A product created on the build tray of a printer is considered not only as a solid with a specific geometry, but also as a new solid material with specific mechanical properties. The research in this area has concentrated on:

− The characteristics of the input material (powders in the case of SLS).
− The geometries of 3D printed objects (including the dimensional tolerances and surface topology).
− The mechanical and rheological properties of 3D printed materials.

Additive manufacturing technologies, especially SLS, are thoroughly described in [23]. The details include theoretical fundamentals, equipment, materials and experimental data. Results of static uniaxial tensile tests for laser sintered polyamide 12 (LS–PA12) are discussed in [24]. The aim of the study was to analyze the behavior of specimens fabricated in three different build directions. The properties considered were Young’s modulus and Poisson’s ratio.

SLS technology has also been applied to create pharmaceutical prototypes, as described in [25]. This technology has been tested to assess its suitability to produce medicines. The article discusses two thermoplastic pharmaceutical polymers: a polyethylene glycol–polyvinyl alcohol graft copolymer, brand name Kollicoat IR, composed of 75% of polyvinyl alcohol and 25% of polyethylene glycol, and a copolymer of methacrylic acid and ethyl acrylate (1:1 ratio), brand name Eudragit L100-55. The medicines have immediate and modified release dosage, respectively. Both polymers were tested with three different loadings of paracetamol (acetaminophen): 5%, 20% and 35%. The process of sintering was facilitated by adding 3% Candurin^®^ gold lustre to each of the acetaminophen powder mixtures. A total of six materials were produced via SLS. The prints (3D printed tablets) were reported to be solid. There was no evidence of drug degradation. These findings indicate that SLS is a universal and practical technology with a potential to be used in the pharmaceutical industry to produce state-of-the-art medicines. The study has largely contributed to the development of 3D printing.

This article focuses on the rheological properties of a polyamide P12-based composite material containing a low percentage of aluminum. Details of the material properties are provided in [19,26,27]. The material is well suited to fabricating medical devices such as orthoses or biomodels. The study involved describing the rheological test results and analyzing them mathematically. Two models were used in the calculations: the five-parameter Maxwell–Wiechert model to determine the stress relaxation of the material, and the five-parameter Kelvin–Voight model to predict its creep behavior. Selecting a mathematical model and fitting it to experimental results is the most difficult part of any research into the rheological behavior of materials.

## 2. Methods and Materials

**Figure 1:**
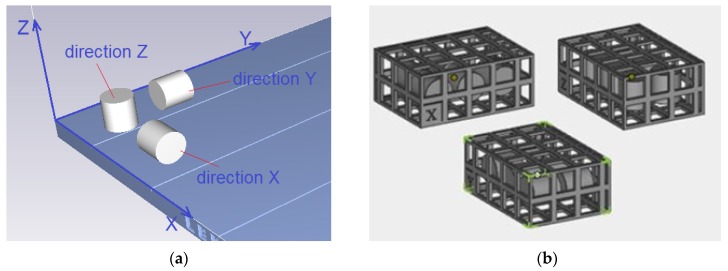
Specimens built in three directions, (**a**) orientation on the build tray, (**b**) arrangement in the protective baskets.

The specimens to be tested were made from a blend of polyamide and aluminum powders, commercially known as Alumide. The specimens were fabricated through SLS using an EOSINT P 760. The light source was a laser with a nominal power of 50 W and a wavelength of 10.6 μm. Alumide is a metallic powder material applied to achieve high rigidity and fine finish. The specimens were cylindrical in shape, with the nominal dimensions being D = 10 mm and H = 10 mm. The solid models were designed in 3D CAD and saved in the * stl. format using the triangulation parameters in the export option: resolution (adjusted), deviation (0.016 mm tolerance), and angle (50 tolerance). Then, the specimen models were virtually oriented on the build tray of the printer, as illustrated in Figure 1.

A layer thickness of 0.12 mm was used to fabricate the cylindrical specimens. The process parameters were selected in accordance with the recommendations of the manufacturer of the Alumide powder. After printing was completed, the specimens and the powder remains were removed from the build tray, and the specimens were prepared for the stress relaxation and creep tests.

The static compressive strength tests were carried out using an Inspekt mini 3 kN universal testing machine produced by Hegewald and Peschke MPT GmbH (Nossen, Germany), equipped with flat compression platens. After the measurement data were acquired, the test parameters were set using Labmaster software incorporated in the Inspekt mini. The specimens, one by one, were placed centrally on the lower platen in the vertical position. Then, the upper platen mounted in the crosshead grip was moved down to be in contact with the flat surface of the specimen.

**Figure 2:**
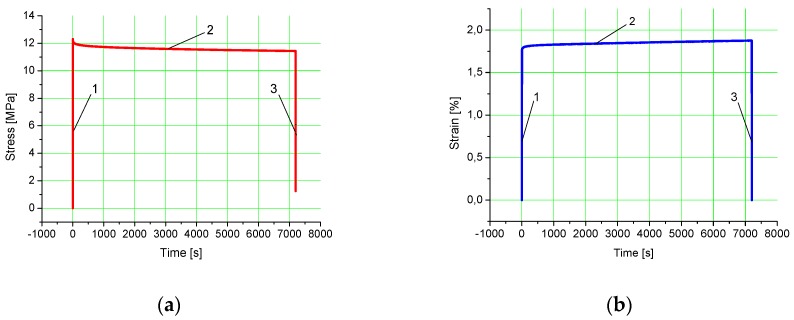
Experimental curves for the specimens SLS fabricated from polyamide-aluminum powder: (**a**) stress relaxation (1—loading represented by the quasi-step function, 2—relaxation, and 3—unloading), (**b**) creep (1—loading represented by the quasi-step function, 2—creep, and 3—unloading).

The first stage of the stress relaxation test involved applying a strain of 5%, with the rate of the compression platen displacement *v* being 0.5 mm/s. In the second stage of the test, the crosshead motion was stopped to apply a constant strain of 5% for a predefined period of time, i.e., 7200 s. There was a decrease in the compressive load and, consequently, a decrease in compressive stresses (stress relaxation), which was illustrated in the form of a curve. In the third stage of the test, the platen returned to the initial (zero) position, which coincided with the unloading of the specimen. An example curve illustrating the whole stress relaxation test is shown in Figure 2a.

The first stage of the creep test consisted in applying a load of 300 N, which corresponded to a stress of approximately 3.82 MPa, with the rate of the compression platen displacement *v* being 0.5 mm/s. After the crosshead motion was stopped, a load of 300 N (a stress of 3.82 MPa) was maintained constant for 7200 s. The gradient of the strain–creep curve was reported to increase slightly. The third stage of the test involved unloading the specimen. The upper platen returned to the initial (zero) position and the test was stopped. An example creep curve is shown in Figure 2b.

The curves in Figure 2a,b are divided into three zones. The first zone or segment *1* represents a rapid increase in load:

− to achieve a displacement of 0.5 mm in stress relaxation tests;
− to achieve a force of 300 N in creep tests.

**Figure 3:**
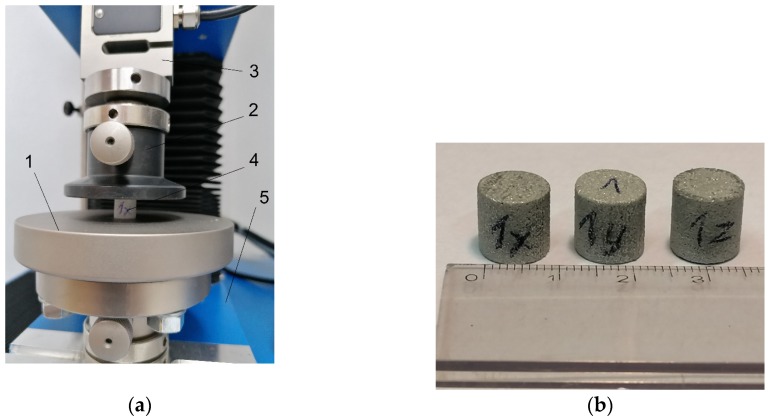
Specimens used in the stress relaxation tests; (**a**) specimen during the test (1—moving lower platen, 2—fixed upper platen, 3—strain gauge load cell, 4—specimen, 5—machine base); (**b**) specimens after the tests.

The rapid increase in load applied to a specimen is represented by the unit step function $\varepsilon (t)=\varepsilon_{0}H(t)$. In theory, the rapid increase in load occurs at an infinitely high rate. This, however, is not possible in reality. In the experiments, load was applied at a relatively high rate; hence the quasi-step function. The second zone of the curve (Segment *2*) is the proper stress relaxation curve (Figure 3a) or the proper creep curve (Figure 3b). Only these curves will be analyzed in this article. The third zone (Segment *3*) illustrates a decrease in load, a return of the upper platen to the output (zero) or the end of test position.

The stress relaxation tests were performed by placing a specimen between two platens of the universal testing machine and compressing it, as depicted in Figure 3a. The specimens after the tests are shown in Figure 3b.

**Figure 4:**
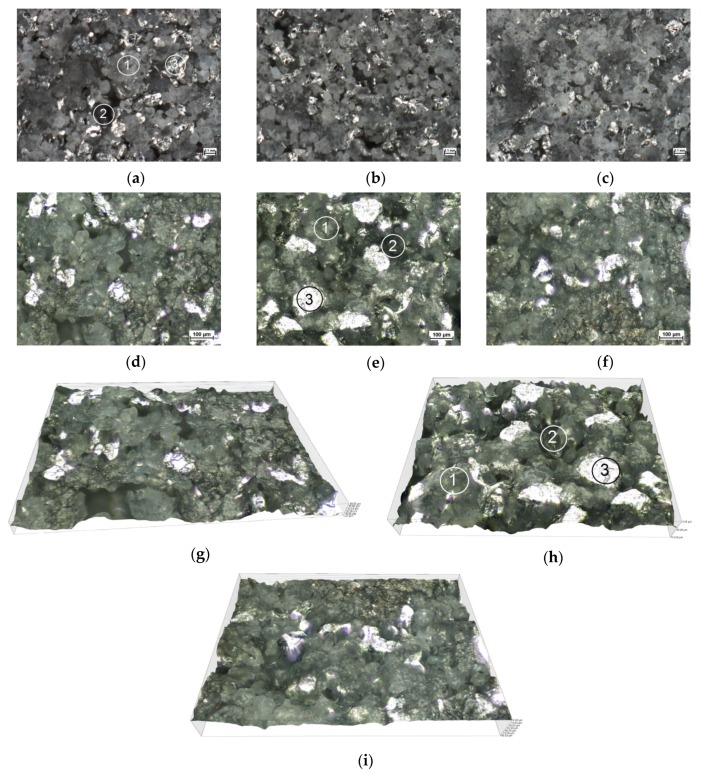
Microscopic images of the specimen surface; (**a**–**c**)—80× magnification; (**d**–**f**)—100× magnification; (**g**–**i**)—3D views, 100× magnification; (**a**,**d**,**g**)—X direction; (**b**,**e**,**h**)—Y direction; (**c**,**f**,**i**)—Z direction; 1—sintered grains of the polyamide powder (P-12); 2—void; 3—aluminum powder grain.

The material of the prints was analyzed using a Nikon Eclipse MA200 microscope equipped with NIS 4.40 AR elements imaging software. Top surfaces of the specimens were examined. The images of the material structure are shown in Figure 4.

The microscopic images reveal that the build direction had no considerable effect on the material structure. It can be seen, however, that the material has voids, with this suggesting that not all the grains of the polyamide powder were fused and bonded. Some of the spherical grains were partially bonded. Aluminum grains are irregular, but they are distributed relatively uniformly in the material structure. The material tested is a typical polyamide-based composite.

## 3. Mathematical Model

Materials able to respond elastically to a rapidly applied load and slowly increasing deformation can be described mathematically by combining two properties: elasticity and viscosity.

This ability can be described linearly using the laws of a Hookean solid and a Newtonian liquid.

The elastic and viscous behaviors of a viscoelastic solid material are mechanically interpreted using two simple single-parameter models: one for a spring and the other for a hydraulic damper.

The simple models are connected in series and/or in parallel to form a system—a mechanical model—describing certain behaviors of a real solid body such as creep and relaxation.

The general equation of the condition describing the two phenomena is:

(1) $$a_{0}\sigma \left(t\right)+a_{1}\dot{\sigma }\left(t\right)+a_{2}\ddot{\sigma }\left(t\right)+\ldots +a_{n}\sigma^{n}\left(t\right)=b_{0}\varepsilon \left(t\right)+b_{1}\dot{\varepsilon }\left(t\right)+b_{2}\ddot{\varepsilon }\left(t\right)+\ldots +b_{n}\varepsilon^{n}\left(t\right)$$

Multi-parameter models may be difficult to use as they require determining a greater number of parameters to calculate the material elasticity and viscosity constants. In this study, a five-parameter model will be considered and Equation (1) will be used to analyze:

- stress relaxation
  (2) $$a_{0}^{r}\sigma^{r}\left(t\right)+a_{1}^{r}{\dot{\sigma }}^{r}\left(t\right)+a_{2}^{r}{\ddot{\sigma }}^{r}\left(t\right)=b_{0}^{r}\varepsilon^{r}\left(t\right)+b_{1}^{r}{\dot{\varepsilon }}^{r}\left(t\right)+b_{2}^{r}{\ddot{\varepsilon }}^{r}\left(t\right)$$
- and creep
  (3) $$b_{0}^{c}\varepsilon^{c}\left(t\right)+b_{1}^{c}{\dot{\varepsilon }}^{c}\left(t\right)+b_{2}^{c}{\ddot{\varepsilon }}^{c}\left(t\right)=a_{0}^{c}\sigma^{c}\left(t\right)+a_{1}^{c}{\dot{\sigma }}^{c}\left(t\right)+a_{2}^{c}{\ddot{\sigma }}^{c}\left(t\right)$$

Since Equations (2) and (3) apply to two different phenomena, the stress, strain and relevant coefficients are distinguished by the superscripts ${}^{r}$ and ${}^{c}$ for stress relaxation and creep, respectively.

Equations (2) and (3) can be solved analytically. The integral Laplace transform is used for this purpose. The general form of this transform for the second and first-order derivatives can be written by the formulas:

(4) $$L\left\{f^{\prime \prime }\left(t\right)\right\}=s^{2}L\left[f\left(t\right)\right]-sf\left(0^{+}\right)-f^{\prime }\left(0^{+}\right)$$

(5) $$L\left\{f^{\prime }\left(t\right)\right\}=sL\left[f\left(t\right)\right]-f\left(0^{+}\right)$$

In this method, the problem is solved using the Heaviside function and the Dirac delta function:

(6) $$H\left(t\right)=\{\begin{array}{c} 1,\;t\geq 0\\ 0,\;t<0 \end{array}$$

(7) $$\delta \left(t\right)=\{\begin{array}{c} +\infty ,\;t=0\\ 0,\;t\neq 0 \end{array}$$

The Laplace transforms of these functions are given by:

(8) L{δ(t)}=1

(9) $$L\left\{H\left(t\right)\right\}=\frac{1}{s}$$

(10) $$L\left\{\delta^{\prime }\left(t\right)\right\}=s$$

Solving this problem analytically will allow us to follow the calculation process and unify the coefficients of Equation (1).

### 3.1. Maxwell–Wiechert Stress Relaxation Model

**Figure 5:**
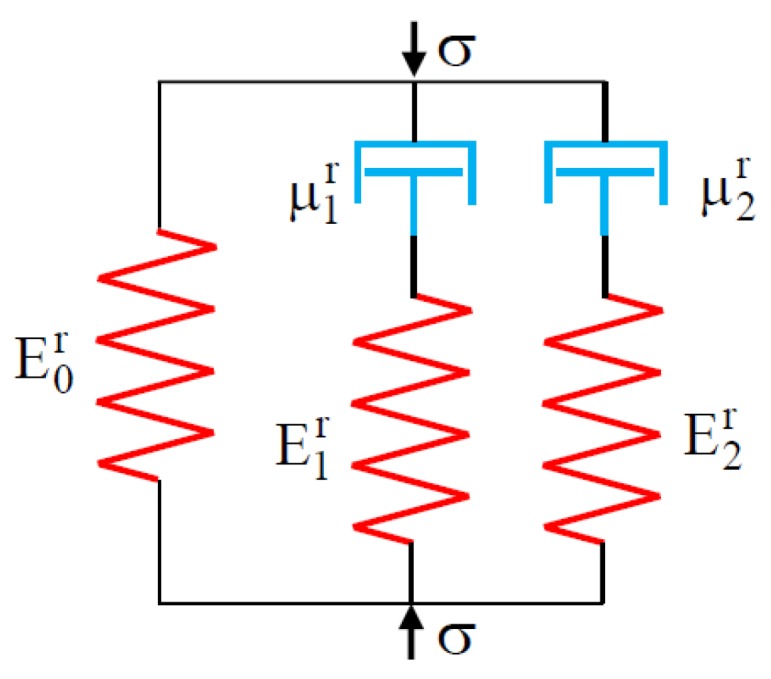
Maxwell–Wiechert stress relaxation model.

The system of springs and dampers used for the five-parameter Maxwell–Wiechert model is illustrated in Figure 5.

Equation (2) can be written as:

(11) $$a_{2}^{r}\frac{d^{2}}{dt^{2}}\sigma^{r}\left(t\right)+a_{1}^{r}\frac{d}{dt}\sigma^{r}\left(t\right)+a_{0}^{r}\sigma^{r}\left(t\right)=b_{2}^{r}\frac{d^{2}}{dt^{2}}\varepsilon^{r}\left(t\right)+b_{1}^{r}\frac{d}{dt}\varepsilon^{r}\left(t\right)+b_{0}^{r}\varepsilon^{r}\left(t\right)$$

Solving this ordinary differential equation requires defining the initial conditions. In the experiment, the initial conditions were: $\sigma^{r}\left(0\right)=\varepsilon_{0}^{r}E_{0}^{r}$ and $\frac{d}{dt}\sigma^{r}\left(0\right)=0$.

The coefficients of Equation (11) are thus as follows:

$$\begin{array}{l} a_{2}^{r}=\mu_{1}^{r}\mu_{2}^{r}\\ a_{1}^{r}=E_{1}^{r}\mu_{2}^{r}+E_{2}^{r}\mu_{1}^{r}\\ a_{0}^{r}=E_{1}^{r}E_{2}^{r} \end{array}\;\begin{array}{l} b_{2}^{r}=\mu_{1}^{r}\mu_{2}^{r}\left(E_{1}^{r}+E_{2}^{r}\right)\\ b_{1}^{r}=E_{1}^{r}E_{2}^{r}\left(\mu_{1}^{r}+\mu_{2}^{r}\right)\\ b_{0}^{r}=E_{0}^{r}E_{1}^{r}E_{2}^{r} \end{array}$$

where: $\varepsilon^{r}\left(t\right)=\varepsilon_{0}^{r}\;H\left(t\right)$, with H(t) being the Heaviside or unit step function. Transforming Equation (11) we get:

(12) $$a_{2}^{r}\frac{d^{2}}{dt^{2}}\sigma^{r}\left(t\right)+a_{1}^{r}\frac{d}{dt}\sigma^{r}\left(t\right)+a_{0}^{r}\sigma^{r}\left(t\right)=\varepsilon_{0}^{r}\;b_{2}^{r}\frac{d}{dt}\delta \left(t\right)+\varepsilon_{0}^{r}\;b_{1}^{r}\delta \left(t\right)+\varepsilon_{0}^{r}\;b_{0}^{r}H\left(t\right).$$

The Laplace transform of the other terms of Equation (12) yields:

(13) $$L\left[\sigma^{r}\left(t\right)\right]=\frac{\frac{\varepsilon_{0}^{r}\;b_{2}^{r}}{a^{r}{}_{2}}s^{2}+\varepsilon_{0}^{r}E_{0}^{r}s^{2}+\frac{\varepsilon_{0}^{r}\;b_{1}^{r}}{a_{2}^{r}}s+\frac{a^{r}{}_{1}}{a_{2}^{r}}\varepsilon_{0}^{r}E_{0}^{r}s+\frac{\varepsilon_{0}^{r}\;b_{0}^{r}}{a_{2}^{r}}}{s\left(s^{2}+\frac{a_{1}^{r}}{a_{2}^{r}}s+\frac{a_{0}^{r}}{a_{2}^{r}}\right)}.$$

The solution of Equation (13) can be written as:

(14) $$L\left[\sigma^{r}\left(t\right)\right]=\frac{A^{r}}{s}+\frac{B^{r}}{\left(s+\frac{1}{\tau_{1}^{r}}\right)}+\frac{C^{r}}{\left(s+\frac{1}{\tau_{2}^{r}}\right)}.$$

After algebraic calculations, we have $A^{r}=\varepsilon_{0}^{r}E_{0}^{r},\;B^{r}=\varepsilon_{0}^{r}E_{1}^{r},\;C^{r}=\varepsilon_{0}^{r}E_{2}^{r}$.

Equation (14) is solved by using the inverse Laplace transform, which gives:

(15) $$\sigma^{r}\left(t\right)=\varepsilon_{0}^{r}\left(E_{0}^{r}+E_{1}^{r}e^{-\frac{t}{\tau_{1}^{r}}}+E_{2}^{r}e^{-\frac{t}{\tau_{2}^{r}}}\right)$$

or, in the general form:

(16) $$\sigma^{r}(t)=\varepsilon_{0}^{r}\left(E_{0}^{r}+\sum_{i=1}^{n}E_{i}^{r}e^{-\frac{t}{\tau_{2}^{r}}}\right)$$

where: $\tau_{1}^{r}=\frac{\mu_{1}^{r}}{E_{1}^{r}}$ and $\tau_{2}^{r}=\frac{\mu_{2}^{r}}{E_{2}^{r}}$.

Equation (3) was solved for creep in a similar way.

### 3.2. Kelvin–Voight Creep Model

**Figure 6:**
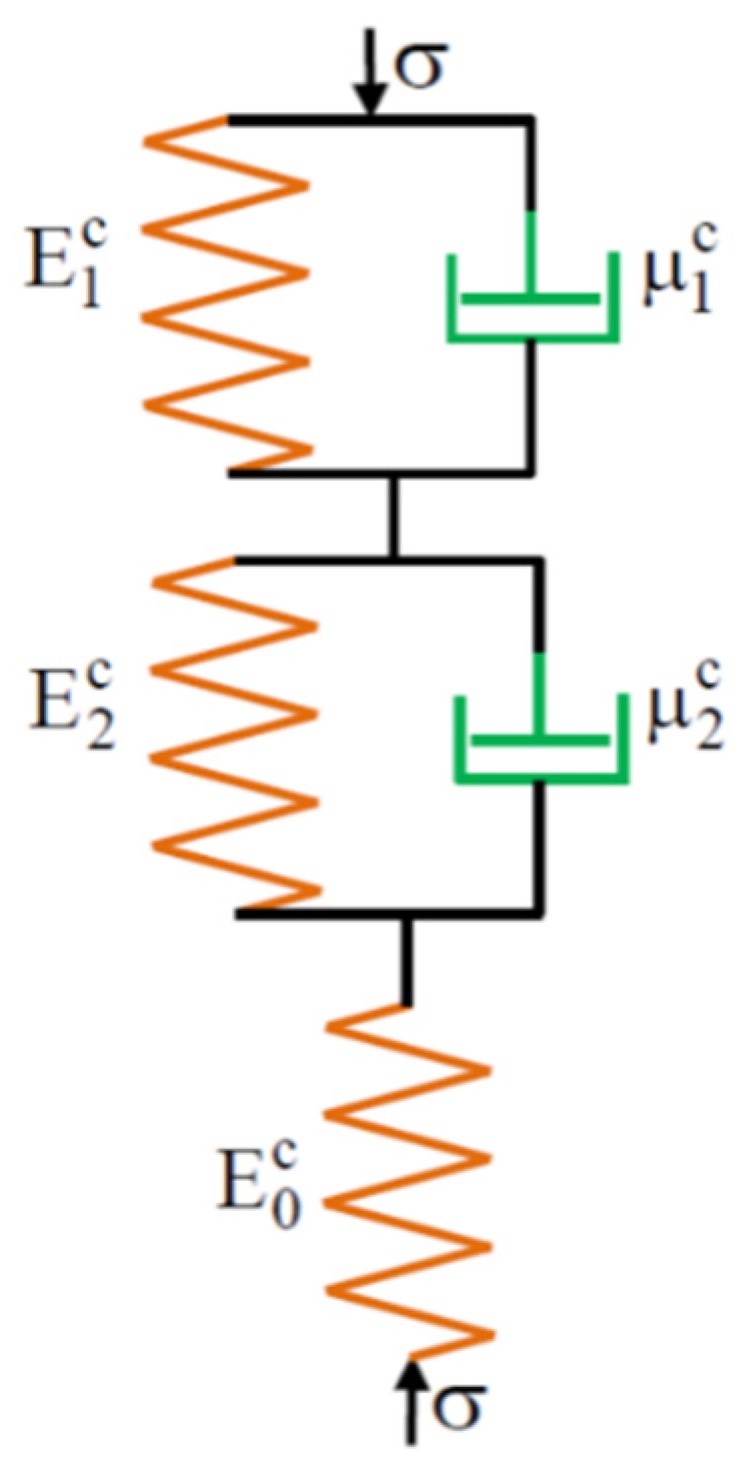
Kelvin–Voight creep model.

The configuration of the springs and dampers used for modelling the material creep is illustrated in Figure 6. In the creep model, when $\sigma^{c}=\sigma_{0}^{c}$, then the stress is constant.

For calculation purposes, Equation (3) is written as:

(17) $$b_{2}^{c}\frac{d^{2}}{dt^{2}}\varepsilon^{c}\left(t\right)+b_{1}^{c}\frac{d}{dt}\varepsilon^{c}\left(t\right)+b_{0}^{c}\varepsilon^{c}\left(t\right)=a_{2}^{c}\frac{d^{2}}{dt^{2}}\sigma^{c}\left(t\right)+a_{1}^{c}\frac{d}{dt}\sigma^{c}\left(t\right)+a_{0}^{c}\sigma^{c}\left(t\right).$$

With the initial conditions being $\varepsilon^{c}\left(0\right)=0$ and $\frac{d}{dt}\varepsilon^{c}\left(0\right)=0$, the coefficients of Equation (17) are:

$$\begin{array}{l} b_{2}^{c}=E_{0}^{c}\mu_{1}^{c}\mu_{2}^{c}\\ b_{1}^{c}=E_{0}^{c}\left(E_{1}^{c}\mu_{2}^{c}+E_{2}^{c}\mu_{1}^{c}\right)\\ b_{0}^{c}=E_{0}^{c}E_{1}^{c}E_{2}^{c} \end{array}\;\begin{array}{l} a_{2}^{c}=\mu_{1}^{c}\mu_{2}^{c}\\ a_{1}^{c}=\left(E_{1}^{c}\mu_{2}^{c}+E_{2}\mu_{1}^{c}+E_{0}^{c}\mu_{2}^{c}+E_{0}^{c}\mu_{1}^{c}\right)\\ a_{0}^{c}=\left(E_{0}^{c}E_{1}^{c}+E_{0}^{c}E_{2}^{c}+E_{1}^{c}E_{2}^{c}\right) \end{array}$$

For the case of simple creep loading, $\sigma^{c}\left(t\right)=\sigma_{0}^{c}H\left(t\right)$. Algebraically, the integral Laplace transform gives:

(18) $$L\left[\varepsilon^{c}\left(t\right)\right]=\varepsilon^{c}\left(s\right)=\frac{\sigma_{0}^{c}\left(a_{2}^{c}s+a_{1}^{c}+a_{0}^{c}\frac{1}{s}\right)}{b_{2}^{c}s^{2}+b_{1}^{c}s+b_{0}^{c}}=\frac{A^{c}}{b_{2}^{c}s}+\frac{B^{c}}{b_{2}^{c}\left(s+\frac{1}{\tau_{1}^{c}}\right)}+\frac{C^{c}}{b_{2}^{c}\left(s+\frac{1}{\tau_{2}^{c}}\right)}$$

where:

$$\tau_{1}^{c}=-\frac{\mu_{1}^{c}}{E_{1}^{c}},\;\tau_{2}^{c}=-\frac{\mu_{2}^{c}}{E_{2}^{c}}\;\mathrm{and}\;A^{c}=\sigma_{0}^{c}\left(\frac{1}{E_{0}^{c}}+\frac{1}{E_{1}^{c}}+\frac{1}{E_{2}^{c}}\right),\;B^{c}=-\sigma_{0}^{c}\frac{1}{E_{1}^{c}}\;\mathrm{and}\;C^{c}=-\sigma_{0}^{c}\frac{1}{E_{2}^{c}}.$$

The inverse Laplace transform of the strain-time function yields

(19) $$\varepsilon^{c}\left(t\right)=\sigma_{0}^{c}\left(\frac{1}{E_{0}^{c}}+\left(\frac{1}{E_{1}^{c}}\left(1-e^{-\frac{t}{\tau_{1}^{c}}}\right)+\frac{1}{E_{2}^{c}}\left(1-e^{-\frac{t}{\tau_{2}^{c}}}\right)\right)\right)$$

or, in the general form:

(20) $$\varepsilon^{c}(t)=\sigma_{0}\left(\frac{1}{E_{0}^{c}}+\sum_{i=1}^{n}\frac{1}{E_{i}^{c}}\left(1-e^{-\frac{t}{\tau_{i}^{c}}}\right)\right)$$

where: $\sigma_{0}^{c}$—constant stress, n—number of basic models, i—consecutive number of the model, $\tau_{i}^{c}$—delay of elasticity of the i-th Kelvin model expressed by:

(21) $$\tau_{i}^{c}=\frac{\mu_{i}^{c}}{E_{i}^{c}}$$

where: $\mu_{i}^{c}$—viscosity of the i-th model and $E_{i}^{c}$—elastic modulus of the i-th model.

Thus,

(22) $$\varepsilon^{c}(t)=\varepsilon_{0}^{c}+\varepsilon_{1}^{c}(1-e^{-\frac{t}{\tau_{1}^{c}}})+\varepsilon_{2}^{c}(1-e^{-\frac{t}{\tau_{2}^{c}}})$$

is the transformed strain-time function.

## 4. Results and Discussion

**Figure 7:**
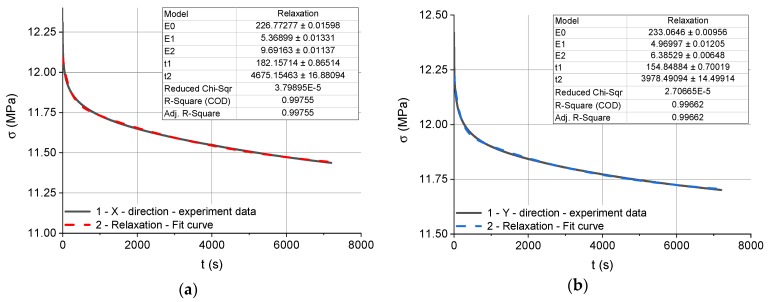

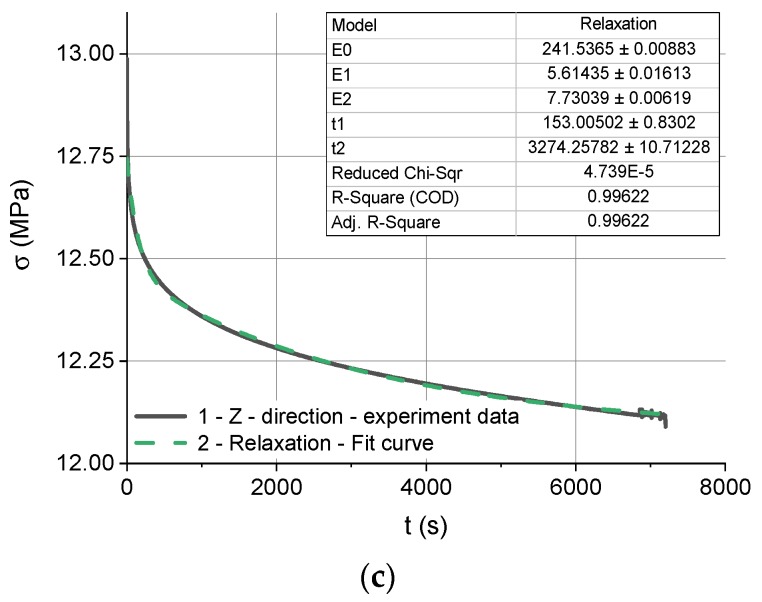
The results of the stress relaxation tests and the best fit curves: 1—experimental stress relaxation curves, 2—stress relaxation curves approximated by Equation (15): (**a**) for the specimens built in the X direction, (**b**) for the specimens built in the Y direction, (**c**) for the specimens built in the Z direction.

Figure 7a–c show the stress relaxation test results and the corresponding best fit curves obtained for the three types of specimen differing in the build direction (X, Y and Z, respectively). The experimental stress relaxation curve is a black continuous line while the best fit stress relaxation curve approximated by Equation (15) is a broken line in red, blue and green. Each figure includes values calculated by the program using a certain number of iterations. These are the parameters $E_{0}^{r},\;E_{1}^{r},\;E_{2}^{r},\;\tau_{1}^{r}$ and $\tau_{2}^{r}$ describing the Maxwell–Wiechert model. There are also approximation errors with the goodness of fit data including the reduced chi-squared *χ*^2^ and the R-squared R^2^.

Each experimental stress relaxation curve was approximated by Equation (15); this required determining the parameters $E_{0}^{r},\;E_{1}^{r},\;E_{2}^{r},\;\tau_{1}^{r}$ and $\tau_{2}^{r}$. The Levenberg–Marquardt algorithm, which is an iterative procedure, was used for curve fitting. The results are given in Table 1.

From the discussion of Equation (15), it is clear that, for t = 0, the stress is:

(23) $$\sigma^{r}(t)=\varepsilon_{0}^{r}\left(E_{0}^{r}+E_{1}^{r}+E_{2}^{r}\right).$$

If we assume that

(24) $$E_{s}^{r}=E_{0}^{r}+E_{1}^{r}+E_{2}^{r}$$

then the stress for *t* = *0* is:

(25) $$\sigma^{r}(0)=\sigma_{s}^{r}=\varepsilon_{0}^{r}\;E_{s}^{r}$$

where: $E_{s}^{r}$—equivalent modulus of elasticity. However, when *t* → *∞*, the limit of Equation (15) is:

(26) $$\sigma^{r}(t_{\infty })=\sigma_{0}^{r}=\varepsilon_{0}^{r}\;E_{0}^{r}.$$

The equivalent elastic moduli and stresses obtained for the different types of specimen at *t* = *0* and *t* → *∞* in the stress relaxation tests are given in Table 2.

The relaxation times $\tau_{1}^{r}$ and $\tau_{2}^{r}$ are the ratios of elastic moduli to dynamic viscosities:

(27) $$\tau_{1}^{r}=\frac{\mu_{1}^{r}}{E_{1}^{r}},\;\tau_{2}^{r}=\frac{\mu_{2}^{r}}{E_{2}^{r}}$$

where: $\mu_{1}^{r}$ and $\mu_{2}^{r}$—dynamic viscosities.

The formulae in Equation (27) and the data in Table 1 were used to calculate the coefficients $\mu_{1}^{r}$ and $\mu_{2}^{r}$ for each specimen type. The values, rounded to the nearest integers, are provided in Table 3.

From the experimental data, it is evident that the polymer-aluminum composite Alumide (all build directions considered) shows slightly lower anisotropy than pure polyamide [19]. The composite material also exhibits lower stress relaxation and creep than pure polyamide P12 additively manufactured through SLS.

**Figure 8:**
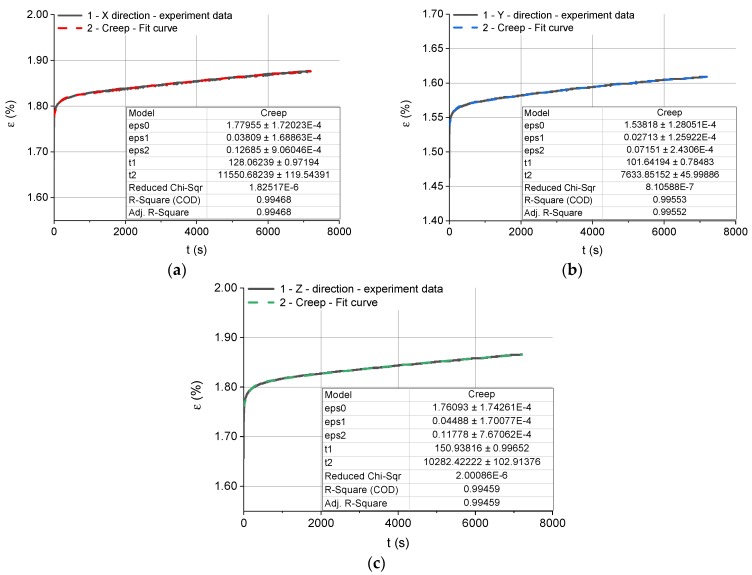
The results of the creep tests with the best fit curves. 1—experimental creep curves, 2—creep curves approximated by Equation (22): (**a**) for the specimens built in the X direction, (**b**) for the specimens built in the Y direction, (**c**) for the specimens built in the Z direction.

The creep test data and the best fit curves are shown in Figure 8.

Each experimental creep curve was approximated by Equation (22); this involved determining the parameters $\varepsilon_{0}^{c},\;\varepsilon_{1}^{c},\;\varepsilon_{2}^{c},\;\tau_{1}^{c}$ and $\tau_{2}^{c}$. The curve fitting was performed using the Levenberg–Marquardt algorithm. The results are provided in Table 4.

The elastic moduli $E_{0}^{c},\;E_{1}^{c}$ and $E_{2}^{c}$, and viscosities $\mu_{1}^{c}$ and $\mu_{2}^{c}$ for the Kelvin–Voight model, illustrated in Figure 4, were calculated from the experimental data using Equation (21). It is important to note that, in the case of creep, the times $\tau_{1}^{c}$ and $\tau_{2}^{c}$ are the retardation times or delays of elasticity of the constituent models. The calculation results obtained for this model are given in Table 5.

From the discussion of Equation (19), it is clear that, at *t* = *0*, the strain is:

(28) $$\varepsilon^{c}(0)=\varepsilon_{0}^{c}=\frac{\sigma_{0}^{c}}{E_{0}^{c}}.$$

However, when *t* → *∞*, the limit of Equation (19) is:

(29) $$\varepsilon^{c}(t_{\infty })=\varepsilon_{Z}^{c}=\sigma_{0}^{c}\left(\frac{1}{E_{0}^{c}}+\frac{1}{E_{1}^{c}}+\frac{1}{E_{2}^{c}}\right)$$

where

(30) $$\frac{1}{E_{z}^{c}}=\frac{1}{E_{0}^{c}}+\frac{1}{E_{1}^{c}}+\frac{1}{E_{2}^{c}}$$

with $E_{z}^{c}$ being the equivalent elastic modulus.

The equivalent elastic moduli and strains obtained for the three different types of specimen at *t* = *0* and *t* → *∞* during the stress relaxation tests are given in Table 6.

The experimental creep data indicate that, at the predetermined level of stress, the same for all specimens, the creep deformation was low; there were no clear differences in creep behavior between the three groups of specimens.

## 5. Conclusions

The specimens fabricated through SLS from Alumide, a polymer-aluminum powder blend, were tested to determine the material stress relaxation and creep according to the build direction. The experimental results show that there are no clear differences in stress relaxation or creep deformation between the three types of specimen (three build directions).

The build direction was observed to affect the material dynamic viscosity in the stress relaxation and creep tests ($\mu_{2}^{r}$ and $\mu_{2}^{c}$, respectively).

The Maxwell–Wiechert model and the Kelvin–Voight model used to describe the stress relaxation and creep behaviors, respectively, were reported to be fully suitable to fit the experimental curves. The experimental and best fit curves coincided well. As a result, it is possible to determine the coefficients of the elastic modulus and dynamic viscosity. The findings can be used to perform a variety of calculations and simulations for any object SLS-fabricated from the material tested.

## Figures and Tables

**Table 1:** Parameters describing the stress relaxation curves for the three types of Alumide specimen.

Build Direction	$E_{0}^{r}$ (MPa)	$E_{1}^{r}$ (MPa)	$E_{2}^{r}$ (MPa)	$\tau_{1}^{r}$ (s)	$\tau_{2}^{r}$ (s)
**X**	226.77	5.37	9.69	182	4675
**Y**	233.06	4.97	6.39	154	3978
**Z**	241.54	5.61	7.73	153	3274

**Table 2:** Equivalent elastic moduli and stresses for *t* = 0 and *t* → *∞*.

Build Direction	$E_{s}^{r}$ (MPa)	$\sigma_{s}^{r}$ (MPa)	$\sigma_{0}^{r}$ (MPa)
**X**	241.83	12.09	11.34
**Y**	244.42	12.22	11.65
**Z**	254.88	12.74	12.08

**Table 3:** Coefficients $\mu_{1}^{r}$ and $\mu_{2}^{r}$ for the different types of specimen.

Build Direction	$\mu_{1}^{r}$ (MPa∙s)	$\mu_{2}^{r}$ (MPa∙s)
**X**	977	45,300
**Y**	765	25,419
**Z**	858	25,419

**Table 4:** Parameters describing the creep curves for the specimens fabricated through SLS from Alumide.

Build Direction	$\varepsilon_{0}^{c}$ (%)	$\varepsilon_{1}^{c}$ (%)	$\varepsilon_{2}^{c}$ (%)	$\tau_{1}^{c}$ (s)	$\tau_{2}^{c}$ (s)
**X**	1.78	0.04	0.13	128	11,550
**Y**	1.54	0.03	0.07	101	7633
**Z**	1.76	0.05	0.12	150	10,282

**Table 5:** Elastic moduli and dynamic viscosities obtained for the Kelvin–Voight model (Figure 8).

Build Direction	$E_{0}^{c}$ (MPa)	$E_{1}^{c}$ (MPa)	$E_{2}^{c}$ (MPa)	$\mu_{1}^{c}$ (MPa∙s)	$\mu_{2}^{c}$ (MPa∙s)
**X**	214.59	9549	2938	1,222,272	33,933,900
**Y**	248.03	12,732	5456	1,285,932	41,645,648
**Z**	217.03	7639	3183	1,145,850	11,781,629,700

**Table 6:** Equivalent elastic moduli and strains at *t* = 0 and *t* → *∞*.

Build Direction	$E_{z}^{c}$ (MPa)	$\varepsilon_{s}^{c}$ (%)	$\varepsilon_{Z}^{c}$ (%)
**X**	195.88	1.78	1.95
**Y**	232.90	1.54	1.64
**Z**	197.91	1.76	1.93

## Nomenclature

$a_{0}$, $a_{1}$, $a_{2}$, $a_{n}$, $b_{0}$, $b_{1}$, $b_{2}$ and $b_{n}$	coefficients of the general equation,
$a_{0}^{r}$, $a_{1}^{r}$, $a_{2}^{r}$, $b_{0}^{r}$, $b_{1}^{r}$ and $b_{2}^{r}$	coefficients of the stress relaxation equation,
$a_{0}^{c}$, $a_{1}^{c}$, $a_{2}^{c}$, $b_{0}^{c}$, $b_{1}^{c}$ and $b_{2}^{c}$	coefficients of the creep equation,
D	specimen diameter,
$E_{0}^{c}$, $E_{1}^{c}$ and $E_{2}^{c}$	elastic moduli for the five-parameter Kelvin–Voight model,
$E_{z}^{c}$	equivalent elastic modulus for the Kelvin–Voight model,
$E_{i}^{c}$	elastic modulus of the i-th model for creep,
$E_{0}^{r}$, $E_{1}^{r}$ and $E_{2}^{r}$	elastic moduli for the five-parameter Maxwell–Wiechert model,
$E_{s}^{r}$	equivalent elastic modulus for the Maxwell–Wiechert model,
$E_{i}^{r}$	elastic modulus of the i-th model for relaxation stress,
H	specimen height,
H(t)	Heaviside function,
δ(t)	Dirac delta function,
$\varepsilon^{c}$	creep strain,
$\varepsilon_{0}^{c},\;\varepsilon_{1}^{c}$ and $\varepsilon_{2}^{c}$	strain for the five-parameter Kelvin–Voight model,
$\varepsilon_{0}^{r}$	predetermined strain for stress relaxation,
$\mu_{i}^{c}$	viscosity of the i-th model for creep,
$\mu_{1}^{c}$ and $\mu_{2}^{c}$	dynamic viscosities in the creep equation,
$\mu_{1}^{r}$ and $\mu_{2}^{r}$	dynamic viscosities in the stress relaxation equation,
$\tau_{i}^{c}$	delay of elasticity of the i-th Kelvin model,
$\tau_{1}^{c}$ and $\tau_{2}^{c}$	retardation times,
$\tau_{1}^{r}$ and $\tau_{2}^{r}$	relaxation times,
$\sigma^{r}$	stress relaxation,
$\sigma_{0}^{c}$	constant stress for creep.
